# On the Nature and Treatment of Apoplexy

**Published:** 1813-11

**Authors:** 


*For the Medical and Physical Journal.*

### *On the* NATURE *and* TREATMENT *of* APOPLEXY.

(Continued from p. 197.)

### *Memoir the Second, by* PORTAL.

THE Observations on the Nature and Treatment of Apoplexy, which I communicated to the Academy of Sciences, and which were printed in their volume for 1781, have proved, that many apoplexies which had been thought serous, were nevertheless sanguineous; and hence conviction must arise, that the signs by which physicians thought to distinguish the sanguineous from the serous apoplexy, such as paleness of the countenance, weakness, smallness and slowness of the pulse, were illusory, and had led physicians to the most baneful practice in the treatment of this malady, since in place of prescribing bleedings, which were necessary, they gave vomits, which could easily prove fatal.

Facts so important, and furnished by anatomy, could not but be useful to medicine. I have become more bold in prescribing bleedings in cases of clearly-marked apoplexy, not in the sanguineous only, in the treatment of which physicians of all ages have agreed, but even in cases in which symptoms the most marked would have indicated to me the existence of serous apoplexy, before I had discovered my error, by the examination of bodies.

Different facts have convinced me that the practice of physicians, who prescribed emetics instead of bleeding in pretended serous apoplexies, was as fatal as the theory on which it was founded was erroneous, of which a judgment may be be formed from some facts I am about to report, and of which I could unfortunately too easily multiply the number.

*Observation the First.*

I was called, in the winter of 1785, to M. Debré, receiver-general, who had been seized with apoplexy. He was very large and very fat. Having been for some time subject to numbness, particularly during the night, he had been advised to lose blood, which he had not done. After dining one day very heartily (for he was a great eater), he suffered a severe attack of apoplexy. Dr. L., who was called, prescribed an emetic without effect. The patient did not vomit, and remained in a profound sleep. I was called to him, and wished to take blood from the jugular, or at least from the foot, but not being permitted, I requested a consultation, and MM. Bouvart and Borie were called in.

We decided that the patient should not only be bled, but that he should be bled several times. He was in fact bled five times in three days, and, in proportion as his vessels were emptied, he found himself in a better state. At first he recovered the movement of the fingers, of the hand, and of the foot of one side only; and successively the movements and sensibility of the limbs and trunk of the same side. His reason returned by remarkable degrees. He spoke, saw, and heard but on one side only, for all the left side continued for a long time paralysed. He continued to take aperients and purgatives in the form of an apozem. He passed to the use of the waters of Balaruc, and at length was enabled to walk; but his left arm remained paralysed for a considerable time, and his tongue was much embarrassed in its movements. A visit to the waters of Bourbonne almost entirely re-established him, and he died three or four years afterwards of a different disease, at a very advanced age.

*Observation the Second.*

In 1790, M. Boutin, treasurer of the marine, aged 60, and very fat, experienced a violent fit of apoplexy at the end of a repast, at which he had eaten largely, and drank wine, spirits, and coffee. An emetic, which had been given him in a very considerable dose before I arrived, produced no effect. I bled him twice in the foot in a very short time; speech returned, and the respiration became more free, but half his body remained paralytic. Blisters, purgative apozems, and ultimately the waters of Balaruc, produced the best effect. The patient insensibly recovered the movement of his limbs, of his inferior extremity first, and then of his arm, which I have observed in all cases recover the last.

 He

He entirely regained his health by the use of the mineral waters, and died three or four years afterwards on the revolutionary scaffold.

*Observation the Third.*

I was called, in 1794, during a severe winter, to M. de Fauveaux, aged 79, who had been attacked with apoplexy. Arriving promptly at his house, I found him senseless, but with a pulse strong and hard, having his respiration stertorous, and his visage red. Apoplexy was here unequivocally marked. The patient was taken as he left table after a repast, at which he had eaten heartily, and drank much wine and other liquors. I was at first inclined, in consideration of his great age, and the fear of indigestion, to administer an emetic; but reflecting on the imminent danger of the patient, and that he had a very short time to live, I thought that I ought not to lose any time by prescribing an emetic, which I had never known to succeed in a similar instance. I therefore bled him in the foot, to the great astonishment of the assistants.

This bleeding was so serviceable that he opened his eyes, moved the fingers of his left hand, and recovered more freedom of respiration.

Deglutition being now more free, I caused him to take an emetic, which produced the most beneficial evacuations. Speech returned to the patient, but he remained deaf, and paralytic of all the right side, from which he recovered in a short time by the use of blisters, of purgative apozems, and of the waters of Balaruc.

This patient was perfectly re-established, and died two years afterwards of a catarrhal putrid fever.

*Observation the Fourth.*

The citizen Gercy was conducted to prison with many other persons who were condemned to death by the revolutionary tribunal: he was acquitted.

A few days afterwards he was seized with apoplexy after a great repast. His respiration was embarrassed; his pulse contracted and small; his visage pale; his limbs very flexible, without motion; and he was generally insensible; yet deglutition remained in a slight degree. I gave him an emetic without effect.

I now did not hesitate to bleed him in the foot, in which I was so successful, that the patient passed almost instantaneously from a state of complete stupor and palsy to the most violent convulsions, which alarmed the attendants, who now considered him much worse than before, but I thought otherwise. On the contrary, I assured them that the citizen

Gercy

Gercy was better, knowing that the convulsions which succeed to paralysis are always less formidable than the profound trance *(assoupissement)*, and that they may even be favorable. I learned this from reading, from clinical observations, and from experiments made on living animals; for I had found when we compressed their brain strongly, that they fell into a profound stupor, and that this stupor diminishing, was succeeded by convulsions.

I advised a second bleeding, which was performed. The convulsions ceased; the patient recovered a little recollection; he pronounced some ill articulated sounds, which in a short time became distinct; but he remained blind. Blisters and purgatives at first produced no effect.

I did not hesitate, however, to declare that I did not despair of the recovery of his sight, if he were conducted to Bourbonne, to Balaruc, or to Barége, persuaded as I was that the compression of the origin of the optic nerves, and of the nerves themselves, the blood-vessels of which I considered as gorged with blood, would be diminished in proportion as that fluid re-entered the circulation, and that vision would consequently be restored, which happened in fact a short time afterwards.

To these observations I could join many others which I have collected, and which would prove that emetics administered to some patients laboring under all the symptoms of serous apoplexy, and to others who were attacked immediately after a copious repast, had been without effect, and that recourse was necessarily had to bleeding; but I shall pass them in silence for the greater brevity.

An emetic administered in such cases is either useless or hurtful, and may even destroy the patient: in effect, if the compression of the nerves in the brain is strong, not only the limbs are paralysed, but the stomach also; then the emetic can exert no action, and is useless. But if any sensibility remains in the stomach, it produces vomiting; but the stomach and muscles of the lower belly, in contracting, force a reflux of blood towards the head; for in persons who vomit, all the parts of the head receive more blood than ordinary, of which we may judge from the redness of the face, the inflammation of the eyes, and bleedings from the nose, which often succeed vomiting: it is not, therefore, surprising that persons have perished under the operation of an emetic.

In this manner it often happens that women perish of apoplexy during parturition, of which I have seen several examples, and a remarkable one in a lady aged 19, who was attacked by a formidable fit, but which was dissipated by numerous bleedings prescribed by my colleague Hallé and myself, and by a successful delivery by citizen Marin.

It

It is an error to suppose that the apoplexy to which old men are so often subject is not sanguineous: the contrary has been proved by the examination of bodies of the most aged persons. Daubenton and Leroy, whose loss the Institute still deplores, died of this species of apoplexy.

The first remained paralysed five days in all his left side. The dissection of his body, at which I assisted with my colleague Cuvier, proved that this great man died from an extravasation of blood into the right ventricle. Daubenton had not been bled: his age; his visage more pale than red; his pulse, which was neither strong nor hard, did not appear to indicate blood-letting.

The citizen Leroy died also of sanguineous apoplexy, and very suddenly. My colleague Lassus and myself assisted at the opening of the body by the citizen Nicolle: we found a quantity of blood extravasated between the cranium and the brain, and much more in the ventricles of this viscus. We were informed that citizen Leroy had bled several times from the nose some days before his death; perhaps he might have escaped had he been bled at that time.

Thinness of apoplectic patients is no reason for not bleeding them; on the contrary, every thing proves that such patients have more blood than others, as Morgagni, Haller, and other physicians, have observed: thus I have bled, with the greatest advantage, patients extremely thin, under a severe apoplectic attack; of this the Marechal de Fitz-James, and the Abbé de Boismont, have furnished me with two examples well known in Paris. Could not every practitioner cite similar instances, if he had prescribed bleeding more boldly? But the apprehension that it may be injurious, sometimes in apoplexies which they suspect from unfaithful symptoms to be serous; sometimes under pretext of indigestion, which they gratuitously suppose to exist, or which they fear without reason; sometimes in relation to old age, which they falsely suppose to be destitute of blood; sometimes in consequence of the thinness of the patient; and, in fine, sometimes in consequence of his weakness, which is often only apparent: all these reasons, I say, prevent practitioners from bleeding in severe apoplexies; and very unfortunately for their patients, who for the most part die, or remain deprived of the use of all their limbs, or at least some of them, or blind, or deaf, or dumb, or with some other misfortune, which might have been prevented by a copious bleeding; which, by depleting the vessels, would have removed the compression on the origin of the nerves, if the congestion of blood was not too great; for it may be sufficient to render the largest bleedings inefficacious, which are now however the less indicated.

P.S. The

P. S. The following is another observation, proving that apoplexy may arise from excess of blood in the brain, although the face may be very pale.

M. Patricot, aged 68, of an ordinary stature, and rather large, who had been for some time deaf, and particularly of late in the right ear, on his return from the country, where he had passed some time, called at the Institute, to speak to one of its members, appearing to enjoy the best health.

He entered the house of Madame de la Rochefoucauld, with whom he lodged; he was called as customary to dinner, and was found in the middle of his chamber senseless, and almost dead. I was summoned instantly, and hastened without delay to his assistance, for the case required it, and M. Patricot was the ancient companion of my studies, and we were united in friendship. I was told that he had been found without sense and motion, particularly of the left side; he had recovered the movement of the right arm when I arrived, and he carried it continually to the right temporal region, as if he there experienced some pain; his respiration was short and sighing, his pulse very weak and undulating, his visage pale and cadaverous. M. Petitbeau, Madame de Rochefoucauld's surgeon, had been called, and had in vain attempted to give him an emetic; I ordered two blisters to his legs, and prescribed an irritating glyster, *(un lavement irritant,)* which the patient could not take. We caused him to inhale some volatile alkali, and endeavored without avail to make him swallow a few drops in some peppermint and orange-flower water; deglutition was intercepted.

Notwithstanding I was convinced that M. Patricot suffered under a most formidable attack of apoplexy, and although so many observations had apprised me, that paleness of the countenance, and weakness of the pulse, were no proofs of its not being sanguineous; and although different observations detailed in my memoirs as those of M. Boutin, of M. de Fourneau, and of others, had convinced me of the utility of bleeding, yet I dared not advise it in this instance; the body of M. Patricot being cold as ice, and his pulse very small and feeble, which had not been the situation of the other patients whom I have mentioned; they, on the contrary, preserved almost their natural heat.

I dared not prescribe bleeding, calling to mind this passage of Aretin's: "Cæterum si frigiditate multa, et torpore vena minime scindenda videatur, subluenda alvus est;"* but I

* De Morb. Anat. lib. i. cap. iv. p. 81.

observed, observed, that if the heat should rise, and the pulse become more full, circumstances very likely to happen, I would advise the application of a dozen leeches to the neck. In effect, some time afterwards the coldness of the body disappeared, its heat returned, and the face of the patient, which had been extremely pale, became very red, crimsoned. Some leeches were placed on his neck, but they obtained little blood. I saw the patient early on the following morning in the last moments of his existence. He died an hour after my visit.

It may easily be imagined that I wished to examine the body, although I was persuaded that the apoplexy was sanguineous; it was necessary to be convinced by an observation, which was made by M. Boyer, my assistant anatomist to the Museum of Natural History, in presence of MM. Petitbeau, de Combettes, and Jean-Paul Martin.

No water was found in the cranium or in the brain, but a small quantity of blood was discovered in the ventricles; and a clot of black blood was lodged partly in an excavation of the right hemisphere, and partly in the right ventricle; all the other viscera were sound.

*Remarks.*—Wepfer also found a clot of blood nearly in the front of one of the hemispheres of the brain, in a woman seventy years of age, who, before the apoplectic attack which destroyed her, had experienced great defect in sight, and a loss of power in the movement of her tongue. The same author, one of the first who wrote on apoplexies from the observation of appearances after death, relates the history of an apoplectic, who, after experiencing a severe attack of gout, of which he was thought cured, was seized with a disease in his chest, which threatened a fatal phthisis pulmonalis; but he suddenly lost sense and motion; paleness of the face, and coldness of the extremities came on, whilst the pulse was strong, full, and frequent, and the respiration laborious. Wepfer did not venture to bleed this patient: "venam in debili tamen et brevi expiraturo tundere non ausus fui."

Nevertheless, *convulsions* supervened, with foaming at the mouth, and stertorous respiration; the body became cold, and the patient died. On opening the body, Wepfer found much blood between the membranes of the brain and in its ventricles: "omnes cruore adimpletos non quarto quidem excepto." The lateral ventricles appeared dilacerated about their base: "ac si nimio cruore distenti rimes egissent." Wepfer could not discover that any vessel of the brain had been ruptured, although more than two pounds of blood were effused.

